# The “Musical Emotional Bursts”: a validated set of musical affect bursts to investigate auditory affective processing

**DOI:** 10.3389/fpsyg.2013.00509

**Published:** 2013-08-13

**Authors:** Sébastien Paquette, Isabelle Peretz, Pascal Belin

**Affiliations:** ^1^Department of Psychology, International Laboratory for Brain Music and Sound Research, Center for Research on Brain, Language and Music, University of MontrealMontreal, QC, Canada; ^2^Voice Neurocognition Laboratory, Institute of Neuroscience and Psychology, University of GlasgowGlasgow, UK; ^3^Institut des Neurosciences de La Timone, Aix-Marseille UniversitéMarseille, France

**Keywords:** music, emotion, auditory stimuli, voices

## Abstract

The Musical Emotional Bursts (MEB) consist of 80 brief musical executions expressing basic emotional states (happiness, sadness and fear) and neutrality. These musical bursts were designed to be the musical analog of the Montreal Affective Voices (MAV)—a set of brief non-verbal affective vocalizations portraying different basic emotions. The MEB consist of short (mean duration: 1.6 s) improvisations on a given emotion or of imitations of a given MAV stimulus, played on a violin (10 stimuli × 4 [3 emotions + neutral]), or a clarinet (10 stimuli × 4 [3 emotions + neutral]). The MEB arguably represent a primitive form of music emotional expression, just like the MAV represent a primitive form of vocal, non-linguistic emotional expression. To create the MEB, stimuli were recorded from 10 violinists and 10 clarinetists, and then evaluated by 60 participants. Participants evaluated 240 stimuli [30 stimuli × 4 (3 emotions + neutral) × 2 instruments] by performing either a forced-choice emotion categorization task, a valence rating task or an arousal rating task (20 subjects per task); 40 MAVs were also used in the same session with similar task instructions. Recognition accuracy of emotional categories expressed by the MEB (*n*:80) was lower than for the MAVs but still very high with an average percent correct recognition score of 80.4%. Highest recognition accuracies were obtained for happy clarinet (92.0%) and fearful or sad violin (88.0% each) MEB stimuli. The MEB can be used to compare the cerebral processing of emotional expressions in music and vocal communication, or used for testing affective perception in patients with communication problems.

## Introduction

With increasing knowledge in the field and new methods to explore the human brain, emotions are no longer too obscure or subjective to be studied scientifically. In neuroscience, many research projects are now entirely dedicated to the study of emotion. Thus, it appears timely to construct a standardized and validated set of stimuli and to make these freely and easily available (www.brams.umontreal.ca/plab_download; http://vnl.psy.gla.ac.uk/resources.php) in order to facilitate the comparability of future studies.

A great amount of work has been achieved in the field of visually perceived emotions, utilizing validated stimuli like the International Affective Picture System and the Ekman faces (Ekman and Friesen, 1978; Lang et al., 1988; Dailey et al., 2001; Ekman et al., 2002), which were designed to portray basic emotions (anger, disgust, fear, happiness, sadness, and surprise as well as a neutral expression). These validated sets of stimuli have provided highly useful tools for the study of brain structures (e.g., amygdala: Adolphs et al., 1994) involved in emotional processing and its developmental trajectory (Charlesworth and Kreutzer, 1973). With the same objectives, an increasing number of studies are being conducted in the domain of aurally perceived emotions, thus calling for validated stimuli sets.

A large part of the research on auditory affective processing has been conducted on speech prosody utilizing words or sentences spoken with various emotional expressions (Monrad-Krohn, 1963; Banse and Scherer, 1996; Buchanan et al., 2000; Kotz et al., 2003; Mitchell et al., 2003; Schirmer et al., 2005; Pell, 2006). Another way to express an emotion vocally is via non-verbal affect bursts (Scherer, 1994; also sometimes called non-verbal interjections: Schröder, 2003). Non-verbal affect bursts are vocal expressions (e.g., screams, laughter) that usually accompany intense emotional feelings. Affect bursts are minimally conventionalized, thus a relatively universal means of spontaneous human communication (see Sauter et al., 2010; Koeda et al., 2013, for cross-cultural studies). They are believed to reflect more of a biological push than a sociological pull (Scherer, 1986); they are closer to the primitive affect expressions of babies and animals than to emotional speech.

Recently, a validated set of auditory affect bursts designed as an auditory counterpart of Ekman faces was recorded and validated by Belin et al. (2008). The so-called Montreal Affective Voices (MAV) consist of a set of short vocal interjections on the vowel /a/ expressing anger, disgust, fear, pain, sadness, surprise, happiness, sensual pleasure, and neutrality. The MAV represent short primitive expressions of these emotions with minimal semantic information, providing useful stimuli for the study of the psychological mechanisms underlying auditory affective processing with minimal interaction with linguistic processes (e.g., Bestelmeyer et al., 2010).

However, vocal affect bursts are not the only means of transmitting auditory emotions. Music is often described as the “language of emotions,” and recent research on basic musical emotions has shown that emotion recognition in music is consistent across listeners (Vieillard et al., 2008). The terms “basic emotions” correspond to a limited number of innate and universal emotion categories (happiness, sadness, anger, fear, and disgust) from which all other emotions can be derived (Ekman, 1982). Moreover, many studies have demonstrated that emotions in music fit Ekman's definition of basic emotions, they are recognized quickly [only a quarter of a second of music; one chord or a few notes (Peretz et al., 1998; Bigand et al., 2005)], early in development (Terwogt and van Grinsven, 1991; Flom et al., 2008), and across different cultures (Balkwill et al., 2004). The latter is even true for cultures without previous exposure to western music (Fritz et al., 2009).

Perception of specific musical emotions (e.g., fear and sadness) can also be lost after damage to the amygdala (Gosselin et al., 2005, 2007), suggesting that damage to the limbic system affects perception of basic musical emotion just as reported for other domains (e.g., vocal expression: Dellacherie et al., 2011; facial expression: Adolphs et al., 1994).

An important question that ensues is why music moves us? Recent studies have shown that certain brain areas [e.g., the striatum (Salimpoor et al., 2011), the amygdala (Gosselin et al., 2007)] are associated with musical emotional processing. These same areas have also been associated with basic biological functions (sex, pain). How can we conceptualize the relationship between music and these neurobiological substrates? One possibility is that music co-opts or invades emotional circuits that have evolved primarily for the processing of biologically important vocalizations [e.g., laughs, screams; Peretz (2010)]. There is currently little experimental data supporting or invalidating the existence of a common musical and vocal channel.

For example, Lima and Castro (2011), demonstrated that musical expertise enhances the recognition of emotions in speech prosody, suggesting that expertise in one domain could translate to the other. Conversely, Thompson et al. (2012), reported that amusics (individual with a pitch perception deficit; Peretz et al., 2002) were also impaired in perceiving emotional prosody in speech.

More specifically, Ilie and Thompson (2006) compared domains by evaluating the effect of manipulating acoustic cues common to both the voice and music [intensity, rate (tempo), and pitch height] on emotional judgments. They found that loud excerpts were judged as more pleasant, energetic and tense compared to soft excerpts, and that fast music and speech were judged as having greater energy than slow music and speech. However, it was also found that tempo and pitch had opposite effects on other emotional scales. Their results support the view that the processing of musical and vocal emotion could utilize common circuitry, but that some of this circuitry might be domain specific.

The existence of domain-specific processes for decoding emotion is consistent with neuropsychological dissociations found between music and language (Peretz and Coltheart, 2003; Omar et al., 2011; Lima et al., 2013). These dissociations could be explained by the fact that musical emotion needs to be actively decoded by the brain based on associations learned via exposure to a musical culture (Peretz et al., 1998; Juslin and Västfjäll, 2008) and past experience with music (Eschrich et al., 2008); since not all musical emotional acoustic parameters are present in emotional vocalizations (e.g., harmony: Juslin and Laukka, 2003), it is possible that these additional cues require additional processing.

Musical and vocal stimuli have both been used to study auditory perceived emotions (Music: Vieillard et al., 2008; Roy et al., 2009; Aubé et al., 2013, Voices: Dalla Bella et al., 2001; Schirmer et al., 2005; Pell, 2006; Fecteau et al., 2007; Belin et al., 2008). Although such stimuli have been quite useful to help exploring aurally perceived emotions in their respective channel, many characteristics set current musical and vocal stimuli apart making them hard to compare in a controlled study. This is especially true for factors such as musical structure (limited by mode or tempo), length, level of complexity as well as the context in which they have been created. The use of pre-existing music can introduce uncontrolled variability of many acoustic parameters, with various demands on attention and memory. Such acoustic and cognitive differences are likely to recruit different neural networks (Peretz and Zatorre, 2005). This is why it is important to create and validate musical stimuli that would be as similar as possible to the MAV to allow for a more proper comparison of aurally (musical and vocal) perceived emotions.

The purpose of the present study is to make available for future research a validated set of brief musical clips expressing basic emotions, designed as a musical counterpart of the MAV. We chose to only include happiness, sadness, and fear because these emotions are among the easiest to recognize from music (Gabrielsson and Juslin, 2003; Juslin and Laukka, 2003; see Zentner et al., 2008, for a more nuanced range of musically induced emotions).

Brief “musical emotional bursts” (MEB) depicting neutral and emotional (happy, sad, and fear) expressions have been recorded from different musicians. The violin and the clarinet were chosen as instruments, not only because they are representative of two different classes of instruments (strings and woodwind) but also because they share important similarities with the voice: “The quasi-vocal quality implied by a seamless progression between notes is a characteristic that can be cultivated in both the clarinet and the violin” (Cottrell and Mantzourani, 2006:33). These recordings were then pre-selected and validated based on listeners' emotion categorization accuracy, as well as on valence and arousal ratings.

## Materials and methods

### Recording

#### Participants

Twenty professional musicians (10 violinists, 10 clarinettists) participated in the recording sessions, after providing written informed consent. They received a compensation of 20$ per h.

#### Procedure

The musicians were first instructed to perform 10 short improvisations with different levels of expressiveness. They were not told in advance what the recording session was about; on the day of the recording they were told one after the other the emotion they were supposed to improvise on, [fear (as if they were scared), happiness, sadness, and neutrality]. They were told their improvisation had to last around a second (they could practice with the metronome), when ready they realized 10 renditions of the emotion. Neutral stimuli were presented just like any other category of stimuli, but characterized as “without emotion.” After improvising, the same musicians were asked to imitate one after another four MAV stimuli depicting fear, happiness, sadness, and neutrality; they could listen to the stimuli as often as they wished. If the emotional category of the musical burst was not clearly recognized by the experimenter (SP) or if the improvisations were too long they were discarded.

The musical bursts were recorded in a sound-treated studio using a TLM 103 large diaphragm microphone Neumann (Georg Neumann, Berlin, Germany) at a distance of approximately 30 cm. Recordings were pre-amplified using a Millennia Media HV-3D preamplifier and digitized at a 44-kHz sampling rate at 24-bit resolution, using Apogee AD16X. Subsequently they were edited into short segments and normalized at peak value (90% of maximum amplitude), using Adobe Audition 3.0 (Adobe Systems, Inc. San Jose, CA).

We ended up with more stimuli than expected, because each musician gave us more excerpts than we asked for. In total, 1505 improvisations [a minimum of 10 × 4 emotions (happy, sad, fear, and neutral) per musician] and 319 imitations of the MAV [a minimum of 4 × 4 emotions (happy, sad, fear, and neutral) per musician] were recorded.

#### Stimulus pre-selection

Improvisations lasting longer than 4 s were excluded. Improvisations or imitations containing an artifact (breathing, vocal sounds, breaking bow hair sounds) were also excluded. In the end, the clearest and most representative stimuli (120 Violin-MEB and 120 Clarinet-MEB) were selected for the validation phase, regardless of their type (improvisation or imitation).

### Validation

#### Participants

Sixty participants (19 males) aged from 19 to 59 years (*M*: 28.8; *SD*: 9.2), with normal hearing participated in an on-line validation test. Each participant gave informed consent and filled out a socio-demographic information questionnaire prior to the judgment phase. Fifteen participants had 6 years or more of musical education and 45 had 5 years or less of training. They were compensated 3£ for their participation.

#### Procedure

Participants were instructed to evaluate each of the 240 MEB and 40 MAV (The MAV were included for comparison with the vocal stimuli). There were 30 violin-MEB, 30 clarinet-MEB and 10 MAV per emotion, and all were presented in a random order. Twenty of the 60 participants performed a four alternative forced-choice identification task “*Please choose the emotion you think this stimulus represents” among* fear, happiness, sadness, and neutrality labels, 20 participants gave arousal ratings “*Please rate on the scale below the perceived arousal of the emotion expressed (from 1 not at all aroused to 9 extremely aroused)*” and another group of 20 participants gave valence ratings “*Please rate on the scale below the perceived valence of the emotion expressed (from 1 extremely negative to 9 extremely positive).*”

## Results

The stimuli (40 violin-MEB and 40 clarinet-MEB) that were best identified (by being categorized in the intended emotion) by the largest amount of participants were selected (10 MEB; 7 improvisations, 3 imitations- per emotion). In the presence of identical ratings, the briefest stimuli were selected. Due to the small number of stimuli in each category, improvisations and imitations were not analysed separately (separate Tables can be found in the Supplementary Material).

Acoustical analyses were also performed to allow users to individually select their stimuli (Supplementary material).

### Emotional categorization

Overall accuracy in the four-alternative emotions categorization task is 85.5% (*SD*: 15.8) for the violin-MEB, 75.4% (23.9) for the clarinet-MEB, and 94.8% (12.1) for the voice-MAV. The average percentage of correct recognition of each intended emotion for the selected stimuli are presented in Table 1. As can be seen, timbre had a greater effect on certain emotional intentions than on others. For example, fear was more difficult to recognize when expressed on a clarinet than on any other timbre.

**Table 1:** **Confusion matrix of emotion recognition for the MEB and MAV**.

	**Intended emotion**	**Forced choice**			
		**Happiness**	**Fear**	**Sadness**	**Neutral**
Violin MEB	Happiness	**76.0 (3.1)**	13.0	4.0	7.0
	Fear	6.5	**88.0 (3.9)**	0.5	5.0
	Sadness	4.0	5.5	**88.0 (3.4)**	2.5
	Neutral	2.0	2.5	5.5	**90.0 (2.9)**
Clarinet MEB	Happiness	**92.0 (2.0)**	2.0	0.5	5.50
	Fear	15.0	**47.5 (4.6)**	13.0	24.5
	Sadness	3.0	9.5	**80.5 (4.1)**	7.0
	Neutral	2.0	3.5	13.0	**81.5 (4.2)**
Voice MAV	Happiness	**98.5 (1.1)**	0.0	1.5	0.0
	Fear	1.5	**93.0 (2.2)**	2.0	3.5
	Sadness	3.5	0.5	**96.0 (1.5)**	0.0
	Neutral	1.0	7.5	0.0	**91.5 (4.5)**

Each row represents the percentage of choices for each emotion in each timbre. Percentage of correct recognition is presented in bold, (SE).

The ANOVA conducted on the recognition scores (see values in bold in Table 1) with Timbre (violin, clarinet, and voice) and Emotion (happiness, sadness, fear, and neutrality) as within-subject factors yielded a main effect of timbre [*F*_(2, 38)_ = 79.51, *p* < 0.001, η^2^ = 0.81] and of emotion [*F*_(3, 57)_ = 6.81, *p* < 0.005, η^2^ = 0.26]; however, they are modulated by a significant interaction between Timbre and Emotion, [*F*_(3.4, 64.4)_ = 16.41, *p* < 0.001, η^2^ = 0.46, corrected Greenhouse-Geisser].

Recognition scores were compared using Tukey's honestly significant difference. Scores averaged across emotions for each timbre were all significantly different (all *p* < 0.005) from one another: voices yielded the highest recognition scores and clarinet the lowest. Comparing emotions, fear was overall significantly (*p* < 0.01) less accurately recognized than all other emotions.

Using binomial tests to determine if the emotions conveyed by each of the 80 stimuli were recognized above chance level (25%), we found that 87.5% (70/80) of the MEB were recognized above chance (*p* < 0.05; bonferroni corrected). Thus, most MEB are effective in expressing an emotion on a musical instrument. Eight of the 10 stimuli that failed to be recognized belonged to the clarinet-fear category; the other two stimuli were from the violin-joy category.

### Emotional ratings

**Figure 1:**
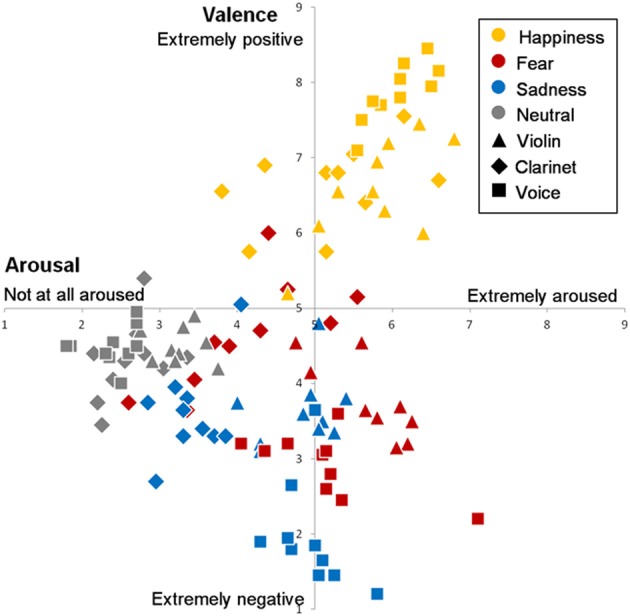
**Valence and arousal ratings for each stimulus played either on violin, clarinet, or voice as a function of the emotional intention**.

The arousal and valence ratings averaged across participants for each stimulus are presented in Figure 1. The individual ratings are provided in the Supplementary Material.

The same ANOVA with Timbre and Emotion as between-subjects factors as the one performed on the recognition scores was computed on the arousal ratings. A main effect of timbre [*F*_(2, 38)_ = 10.05, *p* < 0.001, η^2^ = 0.35] and of emotion [*F*_(3, 57)_ = 33.94, *p* < 0.001, η^2^ = 0.64] were observed; however as previously an interaction between Timbre and Emotion was obtained, [*F*_(6, 114)_ = 5.85, *p* < 0.001, η ^2^ = 0.24].

In general, the clarinet stimuli were judged to be less arousing than the violin and the vocal ones (all *p* < 0.05; by Tuckey's tests), whereas the latter two were judged to be equally arousing (*p* = 0.67). Neutral expressions were overall significantly less arousing (*p* < 0.001) than all other emotions, and happy stimuli were found to be more arousing (*p* < 0.001) than the sad ones.

It is important to note that the stimuli played on a clarinet were rated differently than the violin and vocal stimuli. Happy clarinet stimuli were rated as more arousing than all the other emotions played on clarinet (all *p* < 0.05); [fear was also significantly (*p* < 0.005) more arousing then the neutral stimuli]. In contrast however, the only significant difference for violin and vocal emotional bursts was that neutral stimuli were significantly less arousing (all *p* < 0.01) than all other stimuli.

Regarding valence ratings, we found qualitatively a similar pattern for both the violin and vocal stimuli (Happy > Neutral > Fear > Sad). The clarinet stimuli showed however a slightly different pattern, where fear was rated as being more positive than neural stimuli (Happy > Fear > Neutral > Sad). Again, both a main effect of timbre [*F*_(2, 38)_ = 6.13, *p* < 0.05, η^2^ = 0.24] and of emotion [*F*_(3, 57)_ = 116.65, *p* < 0.001, η^2^ = 0.86] were observed, while the interaction between Emotion and Timbre was again found to be significant [*F*_(6, 114)_ = 31.64, *p* < 0.001, η^2^ = 0.63]. Overall, violin MEB were judged to be less positive than the vocal ones (*p* < 0.005), but globally emotions were significantly different from one another in terms of their valence ratings (*p* < 0.005).

This interaction can be explained by the fact that some differences were observed within timbre. Among the vocal stimuli, the happy ones were judged to be more positive than the neutral ones which were rated as more positive than fear, which in turn was also rated more positively than sadness (all *p* < 0.01). When played on a musical instrument, the happy stimuli were also judged as most pleasant (all *p* < 0.001), whereas only the sad stimuli were rated as significantly more negative than the neutral ones when played on violin (*p* < 0.05), and also as more negative than the stimuli expressing fear played on the clarinet (*p* < 0.005).

## Discussion

Here we validate the MEB—a set of short music clips designed to express basic emotions (happy, sad, fear, and neutral). Despite their short duration (1.6 s on average), the MEB stimuli were correctly categorized by emotion with high accuracy (average recognition score of 80.4%). The highest accuracy was obtained on the violin for stimuli expressing fear and sadness (88%) and on the clarinet for those conveying happiness (92%). Although, the MAV stimuli were best recognized, the newly created MEB were still accurately portraying the desired emotions.

Only three emotions were tested here to allow for direct comparison between basic vocal (MAV) and musical (MEB) emotions. Our limited selection of emotions does limit voice-music comparison, but it is a first step in making that comparison. We acknowledge that there are multiple declinations of positive and negative emotions in the musical and vocal literature, our aim was to use the most easily recognized common to both domains. From a dimensional approach, basic emotions can be distinguished on the dimensions of valence and arousal; variations of these (and other) emotions also differ in valence and arousal and can easily be represented along basic emotions.

The arousal and valence ratings obtained here fit well with this dimensional representation of emotions, with happy stimuli as conveying positive and arousing emotions, fear stimuli as conveying negative and arousing emotions (with the exception of a few clips played on clarinet), sad stimuli as conveying moderately arousing and negative emotions, and the neutral stimuli as conveying an emotional valence that is neither positive or negative with little arousal.

Although the valence scale had a highest rating possible of 9, it is important to note that the maximal average arousal elicited by our stimuli is 6.8 (7.1 for voice), Perhaps the short duration of our stimuli limited their arousing capabilities and could potentially explain the partial overlap in arousal observed in Figure 1 between our two negative emotions (fear, sadness). Also, the fact that the valence scale ranged from “extremely negative” to “extremely positive” (Belin et al., 2008; Aubé et al., 2013), and not from “unpleasant” to “pleasant” could explain why the sad stimuli are differently positioned on the scale than in previous studies (e.g., Vieillard et al., 2008). Nevertheless, our results are still quite similar to those of Vieillard et al. (2008), which were obtained with longer and more conventional musical stimuli (inspired from film music), suggesting that the MEB may tap into similar emotional processes as those evoked by more elaborate film music clips. Yet, the MEB consist of brief expressions and are less likely to involve high-level cognitive mechanisms such as divided-attention and sophisticated knowledge of musical structure than more conventional musical stimuli. The MEB are not limited by tonality or defined by a specific rhythm; they were created as short musical bursts, by professional musicians on their instrument.

Our stimuli can be viewed as a primitive form of musical emotion, situated somewhere in between long musical excerpts from recordings (e.g., Peretz et al., 1998) or short musical segments extracted from these (Dalla Bella et al., 2003; Filipic et al., 2010) and synthesized frequency-modulated tones designed to mimic key acoustic features of human vocal expressions (Kantrowitz et al., 2013). Our novel stimuli were created to be exactly where they are in this spectrum by representing the most basic form of musical emotion that can be closely related to vocal expressions. Although exact replicas of the MAV could have been used instead, by digitally transposing the MAV to another timbre, we chose to produce new recordings in order to keep the stimuli as natural (realistic) as possible.

The timbre, or instrument on which music is played, is known to have an important impact on emotion recognition (Behrens and Green, 1993; Gabrielsson and Juslin, 1996; Balkwill and Thompson, 1999; Hailstone et al., 2009). For example, Hailstone et al. (2009) have found that melodies sound less happy when played on the violin than on other instruments, as we found here. This effect was particularly clear in the imitations of vocal expressions (see Supplementary Material). A range of timbres were used in prior studies (including violin and voice) and each instrument seemed to present its own possibilities and limitations when it came to expressing specific emotions. For instance in our study, we observed that fear was not well recognized when expressed on the clarinet.

Other limitations will also need to be addressed. For example, a forced-choice emotion recognition task was used here, and such tasks can have an impact on statistical analyses, such as increased co-linearity (if one response is chosen, the others are not), which generates artificially high recognition rates (Cornwell and Dunlap, 1994; Frank and Stennett, 2001). This method was selected to facilitate the web-based validation procedure of a large number of stimuli (280), and we believe the technique has served its purpose, as significant differences were observed between the timbres and within each timbre as revealed within the confusion matrix.

In addition, musicians were explicitly asked to imitate vocalizations (3/10 MEB per emotion). Such imitations produced on an instrument with voice-like characteristics may limit the chance to obtain domain-specific responses. In contrast, by using such a setup, finding evidence for domain-specificity would be compelling, even more so if parameters like pitch, emotion recognition scores and valance/arousal ratings are controlled for and used as regressors (Supplementary material) to compensate for the observed differences.

Here we propose a validated set of auditory stimuli designed as a musical counterpart of the MAV to allow a better comparison between auditory (musical and vocal) stimuli designed to convey emotions. We hope that the MEB will contribute to the understanding of emotions across domains and modalities.

### Conflict of interest statement

The authors declare that the research was conducted in the absence of any commercial or financial relationships that could be construed as a potential conflict of interest.
