# Supplementary material for: The “Musical Emotional Bursts”: a validated set of musical affect bursts to investigate auditory affective processing
Source: Front Psychol. 2013 Aug 13;4:509. doi: 10.3389/fpsyg.2013.00509 (PMC3741467; doi:10.3389/fpsyg.2013.00509)
Supplement: Supplementary file 1 [file DataSheet1.DOCX]

(1) Characteristics of violin stimuli. For each stimulus, the duration (second), fundamental frequency (Hertz), ratings and percentage of correct recognition average by stimuli. At the bottom of the tables averages by emotions are presented (Standard deviation).

(2) Characteristics of clarinet stimuli. For each stimulus, the duration (second), fundamental frequency (Hertz), ratings and percentage of correct recognition average by stimuli. At the bottom of the tables averages by emotions are presented (Standard deviation).

(3) Characteristics of vocal stimuli. For each stimulus, the duration (second), fundamental frequency (Hertz), ratings and percentage of correct recognition average by stimuli. At the bottom of the tables averages by emotions are presented (Standard deviation).

(4) Spectral frequency and temporal waveform of the violin stimuli

(5) Spectral frequency and temporal waveform of the clarinet stimuli

(6) For the spectral frequency waveforms of the vocal stimuli, see:

Belin, P., Fillion-Bilodeau, S., Gosselin, F. (2008). The Montreal Affective Voices: A validated set of nonverbal affect bursts for research on auditory affective processing. *Behavior Research Methods, 40*(2), *531-539.*

(7) Percentage of correct identification of the improvised stimuli as a function of timbre and intended emotion (SE).

|  | Violin | Clarinet | Voice |
| --- | --- | --- | --- |
| Happiness | 86.4 (3.5) | 97.9 (1.2) | 98.5 (1.1) |
| Fear | 92.1 (4.3) | 43.6 (5.8) | 93.0 (2.2) |
| Sadness | 90.7 (3.5) | 80.7 (4.8) | 96.0 (1.5) |
| Neutral | 90.0 (3.3) | 80.7 (4.6) | 91.5 (4.5) |

(8) Percentage of correct identification of the imitated stimuli as a function of timbre and intended emotion (SE).

|  | Violin | Clarinet |
| --- | --- | --- |
| Fear | 78.3 (6.1) | 56.7 (6.4) |
| Happiness | 51.7 (7.0) | 78.3 (5.6) |
| Sadness | 81.7 (6.2) | 80.0 (4.5) |
| Neutral | 90.0 (4.3) | 83.3 (5.1) |
